# Silver Nanoparticles Functionalized with Polymeric Substances to Reduce the Growth of Planktonic and Biofilm Opportunistic Pathogens

**DOI:** 10.3390/ijms26093930

**Published:** 2025-04-22

**Authors:** Mădălina Solomon, Alina Maria Holban, Beatrice Bălăceanu-Gurău, Lia Mara Dițu, Adina Alberts, Alexandru Mihai Grumezescu, Loredana Sabina Cornelia Manolescu, Mara Mădălina Mihai

**Affiliations:** 1 Department of Microbiology, Parasitology and Virology, Faculty of Midwives and Nursing, “Carol Davila” University of Medicine and Pharmacy, 020021 Bucharest, Romania; madalina.prd@gmail.com (M.S.); loredana.manolescu@umfcd.ro (L.S.C.M.); 2 Clinical Laboratory of Medical Microbiology, Marius Nasta Institute of Pneumology, 050159 Bucharest, Romania; 3 Department of Botany-Microbiology, Faculty of Biology, University of Bucharest, 030018 Bucharest, Romania; lia_mara_d@yahoo.com (L.M.D.); agrumezescu@upb.ro (A.M.G.); 4 Research Institute of the University of Bucharest, University of Bucharest, 050663 Bucharest, Romania; mara.mihai@umfcd.ro; 5 Department of Oncologic Dermatology, “Elias” Emergency University Hospital, “Carol Davila” University of Medicine and Pharmacy, 020021 Bucharest, Romania; 6 Department of Public Health and Management, “Carol Davila” University of Medicine and Pharmacy, 020021 Bucharest, Romania; adina-magdalena.alberts@rez.umfcd.ro; 7 Department of Science and Engineering of Oxidic Materials and Nanomaterials, Faculty of Applied Chemistry and Materials Science, University Politehnica of Bucharest, Polizu Street No. 1–7, 011061 Bucharest, Romania

**Keywords:** multidrug-resistant bacteria, silver nanoparticles, polymeric substances, planktonic opportunistic pathogens, biofilm forming organisms

## Abstract

The global rise in antimicrobial resistance, particularly among ESKAPE pathogens, has intensified the demand for alternative therapeutic strategies. Silver nanoparticles (AgNPs) have exhibited broad-spectrum antimicrobial activity and represent a promising approach to combat multidrug-resistant infections. This study aimed to synthesize and functionalize AgNPs using various polymeric agents—ethylene glycol (EG), polyethylene glycol (PEG), polyvinylpyrrolidone (PVP), and their combinations—and to evaluate their antimicrobial and antibiofilm efficacy against clinically relevant bacterial strains. AgNPs were synthesized via chemical reduction and functionalized as Ag@EG, Ag@PEG, Ag@EG/PVP, and Ag@PEG/PVP. A total of 68 clinical isolates—including *Staphylococcus aureus*, *Staphylococcus epidermidis*, *Staphylococcus lugdunensis*, *Escherichia coli*, *Klebsiella pneumoniae*, and *Pseudomonas aeruginosa*—were tested. Antimicrobial susceptibility was assessed using disc diffusion and broth microdilution assays, while antibiofilm activity was evaluated via the crystal violet method. Among all tested formulations, Ag@EG/PVP exhibited the highest antimicrobial and antibiofilm activity, with notably low minimum inhibitory concentrations (MIC_50_) and minimum biofilm eradication concentrations (MBEC_50_) for *Ps. aeruginosa* and *K. pneumoniae*. In contrast, AgNPs functionalized with PEG or EG alone showed limited efficacy. Biofilm-forming isolates, particularly *Staphylococcus* spp., required higher concentrations for inhibition. These results highlight the critical role of functionalization in modulating the antimicrobial properties of AgNPs, with Ag@EG/PVP demonstrating potent activity against both planktonic and biofilm-associated multidrug-resistant bacteria. Overall, this study supports further developing AgNPs-based formulations as adjuncts or alternatives to conventional antibiotics, particularly for managing biofilm-related infections. Future research should focus on formulation optimization, safety assessment, and translational potential.

## 1. Introduction

Nanomedicine is focused on the manipulation of materials at the nanoscale (1–100 nm) and exploits different physicochemical characteristics of nanoparticles—particularly their high surface-area-to-volume ratio—to enhance biochemical reactivity and catalytic performance compared to their bulk equivalents [1,2,3,4]. Due to the escalating global challenge of antimicrobial resistance, developing novel and effective therapeutic alternatives has become an essential priority [5,6,7]. Among inorganic nanomaterials, silver nanoparticles (AgNPs) have demonstrated a broad spectrum of antimicrobial, antifungal, antiviral, and anti-inflammatory activities [8,9]. These properties have led to their integration into various biomedical applications such as drug delivery systems, wound dressings, catheter coatings to prevent biofilm formation, and topical formulations such as creams and ointments designed to inhibit opportunistic infections [10,11,12]. As a result, AgNPs have gained prominence as potential agents in addressing multidrug-resistant (MDR) infections, particularly those caused by ESKAPE pathogens—*Enterococcus faecium*, *Staphylococcus aureus*, *Klebsiella pneumoniae*, *Acinetobacter baumannii*, *Pseudomonas aeruginosa*, and *Enterobacter* species [13,14]. These pathogens are known for resisting various antibiotic classes and for their frequent involvement in healthcare-associated infections, posing a substantial global public health threat [13,14].

Beyond their intrinsic antimicrobial capabilities, AgNPs have shown a substantial potential as delivery vehicles for various therapeutic agents, including anti-inflammatory, antioxidant, antimicrobial, and anticancer compounds [15]. Clinically, AgNPs have been incorporated into various products such as wound dressings, catheter and implant coatings, and bone graft materials due to their combined antibacterial and anti-inflammatory effects [15,16,17,18,19,20,21,22,23,24,25,26]. Additionally, their utility has expanded into non-medical domains, including antimicrobial textiles, food packaging, and water purification systems [27,28,29,30,31,32].

Recent advances in nanotechnology have underscored the potential of nanoparticles—particularly silver-based nanomaterials—in combating biofilm-associated infections caused by multidrug-resistant pathogens [8,9]. Several reviews and experimental studies have highlighted the role of AgNPs in targeting biofilm-associated infections caused by multidrug-resistant bacteria. Notably, Kumar et al. emphasized recent nanotechnology-based strategies to inhibit biofilm formation and disrupt microbial communities resistant to antibiotics [33]. Afrasiabi and Partoazar explored how nanoparticle-based delivery systems can modulate gene expression in biofilm-forming bacteria, potentially enhancing treatment precision [34]. Similarly, Sarkar et al. reviewed the antibiofilm potential of nanoparticles against ESKAPE pathogens, underscoring the critical need for novel nanotherapeutics [35]. Proteomics-based insights into the mechanisms of AgNPs’ antibacterial action have also emerged, as shown by Rodrigues et al., who reported key metabolic and structural disruptions induced by silver-based agents [36]. Furthermore, the size of biosynthesized AgNPs has been shown to play a significant role in antibacterial and antibiofilm activity, with smaller particles exhibiting greater efficacy [37].

Other recent advances include innovative combination therapies. Mukherjee et al. described synergistic approaches using nanotechnology to target biofilms of ESKAPE pathogens through enhanced penetration and local delivery [38]. Khairnar et al. demonstrated that conjugating AgNPs with vancomycin improved antimicrobial efficacy against Gram-positive bacteria and enabled better biofilm eradication [39]. Moreover, Szymczak et al. presented a novel strategy combining bacteriophages with silver nanoparticles, showing significantly enhanced antibiofilm performance compared to either therapy alone [40]. Collectively, these studies underscore the therapeutic potential of AgNPs and highlight the need to optimize their functionalization for maximal antimicrobial benefit.

All scientific findings support the continued investigation and optimization of functionalized AgNPs as a versatile platform for managing biofilm-associated and drug-resistant infections. However, limitations such as potential cytotoxicity and environmental impact pose challenges to the therapeutic application of AgNPs [16,17]. These nanoparticles can accumulate in vital organs and may cross biological barriers, including the blood–brain barrier, raising safety concerns [41,42,43,44]. To address these issues, emerging strategies such as conjugation with peptides, antibiotics, or dendrimers aim to enhance efficacy while reducing host toxicity and resistance development [45,46,47,48,49]. Synergistic combinations of AgNPs with antibiotics have shown superior antibacterial activity, enabling lower antibiotic doses and potentially curbing resistance evolution [50,51,52]. Additionally, green synthesis methods using plant extracts or microbial agents offer eco-friendly alternatives with lower cytotoxicity profiles [53,54,55].

Comprehensive biocompatibility assessments and controlled release systems are essential to ensure their safe clinical use. Functionalization with organic or inorganic substances further improves AgNPs biocompatibility and stability. The surface functionalization of AgNPs plays a critical role in determining their stability, dispersion, and interaction with microbial membranes. Polymers such as polyvinylpyrrolidone (PVP), polyethylene glycol (PEG), and ethylene glycol (EG) are widely used to improve nanoparticle biocompatibility and solubility [15,16,17,18,19,20,21]. While prior studies have explored AgNPs functionalized with individual polymers, there remains a significant gap in comparative data evaluating the antimicrobial and antibiofilm performance of AgNPs functionalized with both individual and combined polymers, particularly against biofilm-producing, drug-resistant clinical isolates [15,16,17,18,19,20,21]. Moreover, few studies have systematically assessed these effects using both minimum inhibitory concentrations (MIC) and minimum biofilm eradication concentrations (MBEC) endpoints across a diverse panel of clinically relevant strains.

Our study addresses this gap by synthesizing AgNPs functionalized with PEG, PVP, EG, and their combinations and evaluating their antimicrobial and antibiofilm activity against multidrug-resistant strains—including ESKAPE pathogens—isolated from difficult-to-treat infections. Through side-by-side comparisons using MIC and MBEC assays, we identify the most effective formulation, Ag@EG/PVP, and provide preliminary insights into its potential for clinical translation. This work contributes to the optimization of AgNPs-based antimicrobial strategies and supports their use as viable adjuncts or alternatives to conventional antibiotics in the global effort to combat antimicrobial resistance.

## 2. Results

This study evaluated the antimicrobial and antibiofilm properties of AgNPs functionalized with four different polymeric formulations. A total of 68 bacterial isolates were included, representing clinically relevant infections such as ventilator-associated pneumonia, catheter-associated urinary tract infections, diabetic foot ulcers, central line-associated bloodstream infections, and prosthetic joint infections. The bacterial panel consisted of 11 strains of *E. coli*, 11 strains of *K. pneumoniae*, 23 strains of *Ps. aeruginosa*, 12 strains of *S. aureus*, 6 strains of *S. epidermidis*, and 6 strains of *S. lugdunensis.*

### 2.1. Qualitative Antimicrobial Assessment Using the Disc Diffusion Method

Among all AgNPs formulations tested, only Ag@EG/PVP at 1 mg/mL demonstrated clear antimicrobial activity.

**Figure 1:**
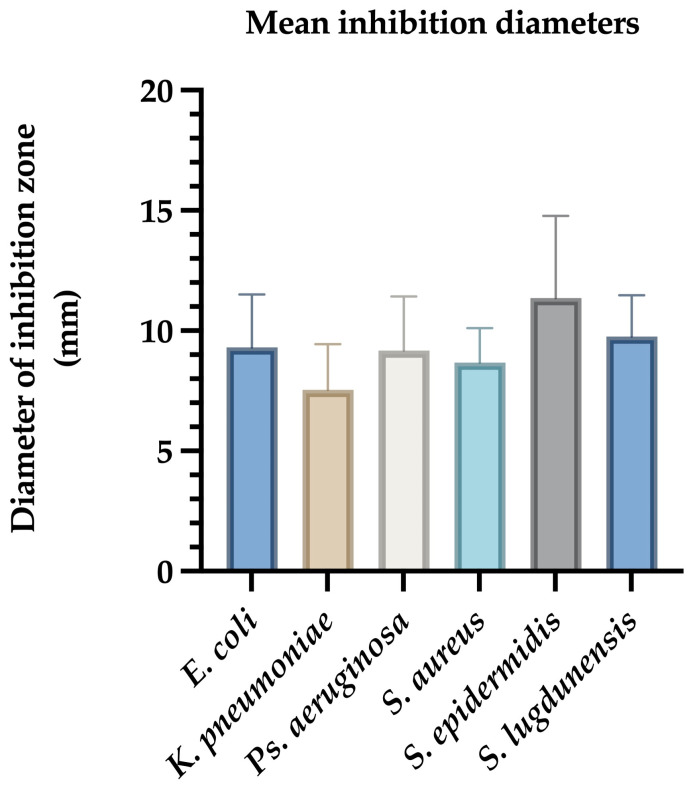
Mean inhibition zone diameters (mm) of Ag@EG/PVP against clinical bacterial isolates. The bar graph illustrates the average diameters of growth inhibition zones produced by AgNPs functionalized with ethylene glycol and polyvinylpyrrolidone (Ag@EG/PVP) against various clinically relevant bacterial strains. Data represent the mean ± standard deviation of triplicate assays conducted in three independent experiments. *S. epidermidis* exhibited the largest mean inhibition zone, indicating high susceptibility, while *K. pneumoniae* showed the smallest zone, consistent with its multidrug-resistant phenotype.

For *E. coli*, inhibition zones ranged from 7 mm to 12.5 mm, with a mean diameter of 9.09 ± 0.85 mm. Reference strains ATCC 25922 and NCTC 13846 showed inhibition zones of 8 mm and 7 mm, respectively (Figure 1).

*K. pneumoniae* isolates exhibited inhibition zones between 4 mm and 11 mm, with a mean diameter of 7.54 ± 1.03 mm (Figure 1).

*Ps. aeruginosa* ATCC 27853 displayed an 8 mm zone, while clinical isolates showed inhibition zones ranging from 6 mm to 15 mm (mean: 9.2 ± 1.58 mm) (Figure 1).

For Gram-positive bacteria, *S. aureus* ATCC 12600 had a 7 mm inhibition zone. Clinical *S. aureus* strains showed inhibition zones ranging from 6 mm to 11 mm (mean: 8.66 ± 0.92 mm). *S. epidermidis* demonstrated the highest average inhibition (8–17 mm, mean: 12.08 ± 1.75 mm), while *S. lugdunensis* showed inhibition zones between 7 mm and 12 mm (mean: 9.75 ± 1.12 mm) (Figure 1).

Ag@EG/PVP demonstrated the most consistent and potent antimicrobial activity across all tested strains. The largest inhibition zone (17 mm) was observed against coagulase-negative *Staphylococci* (CNS), underscoring the superior efficacy of this formulation. Conversely, the least pronounced antimicrobial effect was recorded for *K. pneumoniae*, which may reflect its known multidrug-resistant phenotype. In contrast, the other AgNPs formulations—Ag@PEG, Ag@EG, and Ag@PEG/PVP—exhibited minimal to no inhibitory activity, producing only marginal effects against *K. pneumoniae* with inhibition diameters not exceeding 4–5 mm (Table 1).

### 2.2. Minimal Inhibitory Concentration (MIC)

Ag@EG/PVP yielded the lowest MIC_50_ values: 0.0156 mg/mL for *E. coli* and 0.0039 mg/mL for *K. pneumoniae*, *Ps. aeruginosa*, and coagulase-negative staphylococci (CNS). *S. aureus* strains were less susceptible, requiring higher MIC_50_ values (0.5 mg/mL).

MIC_90_ values also confirmed the superior activity of Ag@EG/PVP, remaining at 0.0039 mg/mL for *Ps. aeruginosa* and 0.0156 mg/mL for *K. pneumoniae*. *S. aureus* again exhibited the highest MIC_90_ values, exceeding 0.5 mg/mL. MIC_50_ and MIC_90_ values were identical for *Ps. aeruginosa*, indicating uniform susceptibility. Other formulations often required concentrations beyond the upper detection limit (>0.5 mg/mL) to achieve inhibition (Table 1).

### 2.3. Minimum Biofilm Eradication Concentration (MBEC)

Biofilm assays showed that *K. pneumoniae* isolates had the lowest MBEC_50_ values (0.0039–0.0156 mg/mL), followed by *Ps. aeruginosa* (0.0039–0.25 mg/mL). Ag@EG/PVP consistently exhibited the most potent biofilm-disruptive effects across species.

Biofilms formed by *Staphylococcus* spp. were more resistant, requiring MBEC_50_ values exceeding 0.5 mg/mL. Interestingly, Ag@PEG was relatively more effective against *E. coli* biofilms (MBEC_50_: 0.0078 mg/mL), whereas other formulations exceeded 0.5 mg/mL.

MBEC_90_ results had the following pattern: values >0.5 mg/mL were required for *Staphylococcus* spp. and *E. coli*, while Ag@EG/PVP achieved MBEC_90_ values of 0.0156 mg/mL for *K. pneumoniae* and 0.0039 mg/mL for *Ps. aeruginosa*. In most cases, MBEC_90_ values were higher than MIC_90_, except in *K. pneumoniae*, where they were equivalent (Table 1).

These findings collectively support the potential of EG/PVP-functionalized AgNPs as potent agents against multidrug-resistant pathogens, particularly in biofilm-associated infections.

## 3. Discussions

Although various AgNPs formulations have been explored for antimicrobial applications, few studies have systematically compared AgNPs functionalized with both individual and combined polymeric agents such as EG, PEG, and PVP. Our study provides a unique comparative framework for assessing the efficacy of these formulations, identifying Ag@EG/PVP as a particularly promising candidate due to its superior performance against both planktonic and biofilm-associated bacterial phenotypes.

Infectious diseases remain a leading cause of global morbidity and mortality, accounting for nearly one-fifth of deaths in 2016 alone [56,57,58]. Respiratory, enteric, and systemic infections caused by bacterial, viral, fungal, and parasitic pathogens impose significant clinical and economic burdens [58,59]. Antibiotics are among the most frequently prescribed drug classes, yet up to 50% of these prescriptions—especially in primary care—are considered inappropriate, further exacerbating AMR [60]. The rapid emergence of MDR pathogens and the scarcity of new antibiotics underscore the urgent need for alternative antimicrobial strategies.

Silver nanoparticles have garnered substantial attention as potent antimicrobial agents, offering broad-spectrum activity against Gram-positive and Gram-negative bacteria [18,61]. AgNPs can be synthesized using top-down approaches such as mechanical milling or bottom-up methods involving physical (e.g., thermoplasma, pyrolytic spray, etc.), chemical (e.g., inverse microemulsion), or biological techniques [16,17]. Among these, chemical reduction is the most widely adopted due to its reproducibility and ability to produce stable colloidal dispersions [16,17]. More recently, biological synthesis using plant extracts or microbial agents has emerged as an eco-friendly alternative, leveraging natural reducing agents such as polyphenols and proteins [62,63,64]. Despite these advancements, challenges remain in fully understanding AgNPs synthesis pathways, optimizing environmental safety, and elucidating molecular interactions with bacterial targets [65].

The antimicrobial activity of AgNPs stems from multiple mechanisms. These include disruption of bacterial membranes, interference with enzymatic processes, and the generation of ROS [66,67,68,69,70,71,72,73,74]. AgNPs increase membrane permeability and structural damage by adhering to the bacterial surface, bind to thiol groups on metabolic enzymes to impair respiration, and interfere with DNA replication and protein synthesis. Additionally, they disrupt quorum sensing and destabilize the extracellular polymeric substance (EPS) matrix within biofilms, leading to disassembly and eradication of mature biofilms [69,75,76]. The efficacy of AgNPs is strongly influenced by physicochemical properties such as particle size, morphology, surface charge, and solubility [77,78]. Notably, spherical or quasi-spherical nanoparticles exhibit greater silver ion release due to their increased surface area, and particles smaller than 10 nm demonstrate heightened antimicrobial activity due to enhanced penetration and reactivity [77,79,80,81,82]. Synergistic use with antibiotics can further enhance antimicrobial effects, reduce required dosages, and potentially mitigate resistance development while minimizing environmental impact [83,84].

Biofilm formation poses a substantial challenge in clinical settings due to its role in conferring high resistance to antibiotics and disinfectants, often up to 1000-fold greater than planktonic cells [85,86,87,88]. Biofilms are a major cause of chronic and nosocomial infections, including respiratory illnesses in cystic fibrosis patients, endocarditis, chronic prostatitis, otitis media, and oral diseases such as periodontitis and caries [85,87,88,89,90,91,92]. Medical devices like catheters, prosthetic valves, and sutures are frequent sites of biofilm colonization, leading to recurrent infections [88,90,91,92,93]. Furthermore, biofilms are implicated in foodborne diseases, forming on surfaces of meat and poultry products, with pathogens like *E. coli*, *Listeria monocytogenes*, and *Salmonella* spp. being notable culprits [94,95,96].

The biofilm matrix, primarily composed of polysaccharides, proteins, and nucleic acids, ensures robust adherence to surfaces and protection against hostile environments [85,97,98,99]. Biofilm development initiates with surface attachment, facilitated by flagella, pili, or lipopolysaccharides, and progresses through microcolony formation regulated by quorum sensing [100,101,102,103]. This process culminates in heightened bacterial resistance and immune evasion, complicating eradication efforts [100,101,102,103].

AgNPs effectively disrupt biofilm formation by inhibiting bacterial adhesion, neutralizing extracellular polymeric substances, and inducing ROS-mediated damage [68,75,85,104,105]. Disruption of bacterial cytoskeletal proteins such as MreB and interaction with cell membranes lead to morphological alterations and cell lysis [68,75,104,105]. Intracellularly, AgNPs bind to thiol groups of enzymes, impairing respiration and metabolism, and interact with DNA, affecting replication and transcription processes [70,76]. Additionally, AgNPs interfere with phosphorylation pathways essential for bacterial growth and can induce apoptosis via p53 and caspase3 activation [67,75]. Notably, the smaller the nanoparticles, the greater their antimicrobial efficacy due to enhanced cellular uptake [16].

Numerous studies have confirmed the antibiofilm potential of AgNPs. For instance, significant inhibition of *S. aureus* and *E. coli* was observed at MIC values of 100 µg/mL, with marked morphological damage [84]. AgNPs (8.3 nm) inhibited *Ps. aeruginosa* PAO1 biofilms at concentrations as low as 4–5 µg/mL [106]. In resistant *Ps. aeruginosa* strains, 20 µg/mL AgNPs reduced biofilm formation by up to 67% [17]. Furthermore, AgNPs suppressed quorum-sensing-regulated virulence factors in *Chromobacterium violaceum* and *Ps. aeruginosa*, highlighting their broad-spectrum activity [15].

Advanced analyses, including SEM and TMT-labeled proteomics, revealed that AgNPs disrupt biofilm architecture, impair bacterial motility and adhesion, trigger oxidative stress responses, and downregulate key metabolic pathways [107]. AgNPs synthesized by *Cedecea* sp. demonstrated strong bactericidal and antibiofilm effects, with notable stability and efficacy against *E. coli* and *Ps. aeruginosa* [108].

In addition to demonstrating efficacy, our findings contribute to the early-stage optimization of AgNPs functionalization strategies. By integrating MIC and MBEC assessments across a diverse panel of MDR clinical isolates—many of which belong to the ESKAPE group—we provide data that may inform the rational design of AgNPs-based therapeutics. These insights are particularly relevant for developing adjunct or alternative therapies aimed at combating the growing threat of antimicrobial resistance in hospital-acquired infections.

In our study, we examined the efficacy of AgNPs formulations against several clinically relevant MDR pathogens. *S. aureus* and coagulase-negative staphylococci such as *S. epidermidis* and *S. lugdunensis* are major contributors to nosocomial and device-associated infections. These organisms, although typically part of the skin microbiota, can become pathogenic in immunocompromised individuals or when the epithelial barrier is disrupted [109,110,111,112]. Their virulence is mediated by surface adhesins, enzymes, and toxins and is particularly enhanced by their ability to form biofilms, which enable persistence on abiotic surfaces and lead to treatment failure and recurrence [111,113,114,115,116]. Methicillin-resistant *S. aureus* strains further complicate therapy, often necessitating last-line agents such as linezolid or vancomycin [117,118]. Infections like hidradenitis suppurativa and chronic abscesses are frequently associated with biofilm-producing *Staphylococcus* species [119,120,121,122]. Our findings confirm previous reports that AgNPs can effectively disrupt staphylococcal biofilms [113,115,116,123,124,125], with *S. epidermidis* showing the highest susceptibility to Ag@EG/PVP in both diffusion and MBEC assays. This supports its potential as a targeted strategy against biofilm-associated staphylococcal infections.

*Acinetobacter baumannii* is a Gram-negative opportunist known for its exceptional genomic plasticity and ability to accumulate diverse resistance determinants. The World Health Organization designates carbapenem-resistant *A. baumannii* as a critical priority pathogen [126,127]. The clinical isolates in our study harbored OXA-type carbapenemases (e.g., OXA-51, OXA-40, etc.) and aminoglycoside resistance genes such as *aac(6′)-Iad*, reflecting its high resistance burden [128,129,130,131,132]. Although it was not the most susceptible species to AgNPs in our assays, its inclusion remains essential given its notorious biofilm formation and limited treatment options. Continued testing of AgNPs formulations, particularly Ag@EG/PVP, is warranted to assess efficacy against persistent and resistant strains.

*Pseudomonas aeruginosa* is a major cause of ventilator-associated pneumonia, catheter-related infections, and chronic wound infections. Its resistance mechanisms include the production of carbapenemases (e.g., VIM-2 and IMP-13) and overexpression of multidrug efflux pumps like MexAB-OprM [125,133,134,135,136,137,138]. Moreover, it forms robust, structured biofilms that impede antibiotic penetration and alter bacterial metabolic states, reducing treatment efficacy [125]. Our study demonstrated that Ag@EG/PVP exhibited excellent antibiofilm and antimicrobial activity against *Ps. aeruginosa*, consistent with previous work on AgNPs-mediated disruption of biofilms and quorum sensing [138,139,140]. These findings highlight its relevance in combating *Pseudomonas*-related infections, particularly in intensive care settings.

*Klebsiella pneumoniae* presents a formidable clinical challenge due to its ability to acquire and disseminate resistance genes through horizontal gene transfer. In our isolates, extended-spectrum β-lactamase production and carbapenemase expression (e.g., NDM and OXA-48) were frequently observed, mirroring global resistance patterns [139,140,141,142]. This species is also known for its capacity to form dense biofilms on mucosal surfaces and medical devices. Interestingly, *K. pneumoniae* demonstrated the lowest MBEC_50_ values in our assays, indicating that Ag@EG/PVP is particularly effective in eradicating its biofilms. This aligns with the existing literature on AgNPs-mediated biofilm disruption in Enterobacteriaceae [124,139,140,141,142].

The high antimicrobial and antibiofilm efficacy observed with Ag@EG and Ag@PEG/PVP formulations underscores their therapeutic potential. MIC values were consistent with those required for biofilm eradication, reinforcing their activity against planktonic and sessile forms. Ag@EG/PVP in particular emerged as the most effective formulation, especially against *K. pneumoniae* and *S. epidermidis*. The enhanced performance is likely due to the synergistic effects of EG and PVP. PVP serves as a steric stabilizer, preventing aggregation and enhancing colloidal stability, while EG, as a polyol and reducing agent, promotes uniform nanoparticle size and improves aqueous dispersion [143,144]. This combination supports a controlled and sustained release of Ag^+^ ions—the primary antimicrobial component [145]. Furthermore, the increased surface functionalization provided by these polymers enhances nanoparticle interaction with bacterial membranes, promoting oxidative stress, membrane disruption, and inhibition of vital cellular processes [145].

The reproducibility of our findings was ensured through the use of multiple independent replicates and triplicate assays. Antimicrobial and antibiofilm results were consistent across all tested strains, and statistical analysis confirmed the significant superiority of Ag@EG/PVP compared to other formulations (*p* < 0.05). These results support the continued investigation of AgNPs-based strategies for clinical use, including wound healing, orthopedic implants, and cardiovascular device coatings [15,16,17]. Our study contributes to the growing evidence that polymer-functionalized AgNPs—particularly those utilizing combined stabilizers—represent a viable path forward in the fight against multidrug-resistant, biofilm-forming pathogens.

## 4. Materials and Methods

### 4.1. Synthesis and Functionalization of Silver Nanoparticles

AgNPs were synthesized and functionalized using four different formulations containing either individual or combined polymeric agents, according to previous published results: EG, PEG, and PVP. The resulting formulations were designated as Ag@EG, Ag@PEG, Ag@EG/PVP, and Ag@PEG/PVP [146,147].

To synthesize AgNPs, 1 g of AgNO3 was dissolved in 300 mL of ultrapure water. Separately, 20 g of NaOH was dissolved in 400 mL of ultrapure water, followed by the addition of one of the following: 3 g EG, 3 g PEG, 1.5 g EG + 1.5 g PVP, or 1.5 g PEG + 1.5 g PVP. Each mixture was stirred continuously at 80 °C. The silver nitrate solution was gradually added dropwise into each polymer solution under constant stirring to facilitate nanoparticle formation. The suspensions were then vacuum-filtered, washed three times with sterile distilled water, and air-dried. The concentration of AgNPs was adjusted to 1 mg/mL in all biological assays.

All inhibition zone measurements presented in this study refer to diameters and are reported in millimeters (mm). Values are expressed with a single decimal place, consistent with the precision of the measuring instrument used.

### 4.2. Bacterial Strains and Growth Conditions

A total of 68 clinical bacterial isolates were included in this study. These were obtained from hospitalized patients diagnosed with infections that are challenging to treat, including urinary tract infections, respiratory infections, and wound-associated infections. Strains were collected from Romanian healthcare settings, where the prevalence of MDR pathogens is high and contributes to elevated morbidity and mortality rates.

The bacterial panel comprised *E. coli* (n = 11), *K. pneumoniae* (n = 11), *Ps. aeruginosa* (n = 23), *S. aureus* (n = 12), *S. epidermidis* (n = 6), and *S. lugdunensis* (n = 6). MDR profiles were confirmed based on resistance to at least three distinct antibiotic classes. Isolates were initially characterized for antimicrobial resistance and virulence factors to ensure clinical relevance and strain diversity.

Antibiotic susceptibility testing was performed using disc diffusion or broth microdilution methods, and resistance patterns were interpreted according to CASFM/EUCAST guidelines (Table S1). The antibiotic discs used for each bacterial species were as follows:

- *Staphylococcus* spp.: cefoxitin (FOX) 30 µg, gentamicin (CN) 10 µg, erythromycin (E) 15 µg, clindamycin (DA) 2 µg, quinupristin-dalfopristin (QD) 15 µg, norfloxacin (NOR) 10 µg, linezolid (LZD) 10 µg, fusidic acid (FD) 10 µg, cotrimoxazole (SXT) 1.25 µg, rifampicin (RD) 5 µg, kanamycin (K) 30 µg, penicillin G (P) 6 µg, and tetracycline (TE) 30 µg;
- *E. coli* and *K. pneumoniae:* amoxicillin/clavulanic acid (AMC) 20/10 µg, amoxicillin (AML) 20 µg, ticarcillin (TIC) 75 µg, piperacillin (PRL) 100 µg, imipenem (IPM) 10 µg, cefotaxime (CTX) 5 µg, cefoxitin (FOX) 30 µg, ceftazidime (CAZ) 10 µg, ticarcillin/clavulanic acid (TIM) 75/100 µg, ciprofloxacin (CIP) 5 µg, levofloxacin (LEV) 5 µg, nalidixic acid (NA) 20 µg, aztreonam (ATM) 30 µg, norfloxacin (NOR) 10 µg, and moxifloxacin (MOX) 5 µg;
- *Ps. aeruginosa*: imipenem (IPM) 10 µg, meropenem (MEM) 10 µg, aztreonam (ATM) 30 µg, cefepime (FEP) 30 µg, ceftazidime (CAZ) 10 µg, ticarcillin/clavulanic acid (TIM) 75/100 µg, ticarcillin (TIC) 75 µg, fosfomycin (FOT) 200 µg, and ceftazidime/avibactam (CZA) 10/4 µg.

Reference strains used for quality control included *S. aureus* subsp. *aureus* Rosenbach ATCC^®^ 12600™, *E. coli* ATCC^®^ 25922™, *E. coli* NCTC 13846, and *Ps. aeruginosa* ATCC^®^ 27853™.

All isolates were stored at −80 °C in nutrient broth supplemented with 20% glycerol. Before use, strains were subcultured on nutrient agar and incubated at 35 °C for 24 h. Bacterial suspensions were prepared in 0.9% saline and adjusted to a 0.5 McFarland turbidity standard (approximately 1–3 × 10⁸ CFU/mL).

### 4.3. Qualitative Antimicrobial Activity: Disc Diffusion Assay

The antimicrobial activity of each AgNPs formulation was evaluated using a modified disc diffusion assay. Mueller–Hinton agar plates were inoculated with the bacterial suspensions using sterile swabs in three directions at 45° angles, following CLSI guidelines. A 5 µL volume of each AgNPs suspension (1 mg/mL) was spotted on the agar surface. Plates were allowed to sit for 15 min to permit diffusion and were then incubated at 37 °C for 20 h. Inhibition zones were measured in millimeters. Each isolate was tested in triplicate across three independent experiments to ensure reproducibility.

### 4.4. MIC Determination

MICs were assessed via broth microdilution in 96-well plates. An initial AgNPs concentration of 5 mg/mL was serially diluted two-fold to obtain concentrations ranging from 0.5 mg/mL to 0.0039 mg/mL. A volume of 15 µL of bacterial suspension was added to each well containing 135 µL of Mueller–Hinton broth and AgNPs. Plates were incubated for 24 h at 37 °C without shaking. MICs were recorded based on visible turbidity and confirmed by spectrophotometric absorbance readings at 620 nm. Assays were performed in triplicate and repeated independently three times.

### 4.5. Evaluation of AgNPs on Biofilm Formation

Biofilm inhibition was quantified using a crystal violet assay. AgNPs were serially diluted as described above. A 15 µL aliquot of bacterial suspension was added to 135 µL of the AgNPs dilution in a 96-well polystyrene microplate. Plates were incubated at 37 °C for 24 h. Wells were gently washed with sterile water to remove planktonic cells, fixed with cold methanol for 5 min, stained with 1% crystal violet for 20 min, and rinsed. The bound dye was solubilized using 33% acetic acid, and absorbance was measured at 492 nm. All experiments were performed in triplicate and repeated independently on three occasions.

### 4.6. Statistical Analysis

All data are presented as mean ± standard deviation (SD). Statistical analysis was conducted using GraphPad Prism (v9.5) and IBM SPSS Statistics (v27). One-way ANOVA followed by Tukey’s post hoc test was used for multiple group comparisons, while two-group comparisons employed Student’s *t*-test. A *p*-value of <0.05 was considered statistically significant. The normality of data distribution was assessed prior to analysis.

The number of clinical isolates was selected to ensure sufficient biological variability across species and infection types, reflecting the microbial diversity encountered in clinical practice. Triplicate assays and independent repetitions were employed to enhance statistical power and experimental reliability.

### 4.7. Methodological Considerations and Study Limitations

One limitation of this study is the absence of unmodified AgNPs as a comparative control group. Although sterile distilled water was used as a negative control, future work should include bare AgNPs to delineate better the impact of polymeric functionalization on antimicrobial and antibiofilm efficacy.

In addition, dynamic light scattering (DLS) and zeta potential measurements were not performed. These parameters are essential for assessing nanoparticle size distribution and surface charge, which influence colloidal stability, cellular uptake, and bioactivity. Future studies should include comprehensive physicochemical characterization to better understand the relationship between nanoparticle structure and antimicrobial performance.

Furthermore, all experiments were conducted in vitro. While these findings offer valuable insight into AgNPs antimicrobial and antibiofilm potential, in vivo studies are necessary to evaluate biodistribution, toxicity, pharmacokinetics, and therapeutic relevance. Animal models and preclinical trials are recommended for future investigations to support clinical translation.

## 5. Conclusions

Our study demonstrated that AgNPs functionalized with polymeric agents exhibit significant antimicrobial and antibiofilm efficacy, particularly when coated with EG and PVP. Among 68 multidrug-resistant clinical isolates, Ag@EG/PVP consistently outperformed other formulations across both planktonic and biofilm conditions, notably against *Ps. aeruginosa* and *K. pneumoniae.*

Our findings underscore the importance of surface functionalization in enhancing the bioactivity of AgNPs and support the development of polymer-modified nanomaterials as adjuncts or alternatives to conventional antibiotics. The demonstrated dual efficacy against resistant pathogens and biofilms positions Ag@EG/PVP as a promising candidate for biomedical applications, including wound dressings, implant coatings, and anti-infective surfaces. Future studies should focus on in vivo validation, toxicity profiling, and formulation strategies to advance clinical translation.

## Figures and Tables

**Table 1:** Comparative analysis of antimicrobial and antibiofilm activity of AgNPs functionalized with different polymeric substances against clinical bacterial isolates. This table summarizes the MIC_50_, MIC_90_, MBEC_50_, and MBEC_90_ values for five clinically relevant bacterial strains (*E. coli*, *K. pneumoniae*, *Ps. aeruginosa*, *S. aureus*, and coagulase-negative *Staphylococci* (CNS)) treated with AgNPs functionalized with PEG, EG, PEG/PVP, and EG/PVP. The results demonstrate that Ag@EG/PVP consistently exhibited the lowest MIC and MBEC values across both Gram-negative and Gram-positive strains, particularly for *K. pneumoniae* and *Ps. aeruginosa*, where MIC_50_ and MBEC_50_ reached as low as 0.0039 mg/mL. In contrast, all other formulations showed significantly higher values, often approaching or exceeding 0.5 mg/mL. Notably, *S. aureus* and CNS exhibited the highest resistance to biofilm eradication, with MBEC_90_ values >0.5 mg/mL across all nanoparticle types.

Bacterial Strain	MIC 50 (Ag@PEG)	MIC50 (Ag@EG)	MIC 50 (Ag@PEG/PVP)	MIC 50 (Ag@EG/PVP)	MIC 90 (Ag@PEG)	MIC90 (Ag@EG)	MIC 90 (Ag@PEG/PVP)	MIC 90 (Ag@EG/PVP)	MBEC 50 (Ag@PEG)	MBEC 50 (Ag@EG)	MBEC 50 (Ag@PEG/PVP)	MBEC 50 (Ag@EG/PVP)	MBEC90 (Ag@PEG)	MBEC 90 (Ag@EG)	MBEC 90 (Ag@PEG/PVP)	MBEC 90 (Ag@EG/PVP)
*E. coli*	0.5	0.5	0.25	0.0156	>0.5	>0.5	0.5	0.03125	0.5	0.5	0.5	0.0078	>0.5	>0.5	>0.5	>0.5
*K. pneumoniae*	0.5	0.5	0.25	0.0039	0.5	0.5	0.5	0.0156	0.5	0.5	0.5	0.0039	0.5	0.5	0.5	0.0156
*Ps. aeruginosa*	0.25	0.25	0.125	0.0039	0.5	0.5	0.25	0.0039	0.25	0.25	0.125	0.0039	>0.5	>0.5	>0.5	0.0039
*S. aureus*	0.5	0.5	0.5	0.5	0.5	0.5	0.5	0.5	>0.5	>0.5	>0.5	>0.5	>0.5	>0.5	>0.5	>0.5
CNS	0.5	0.5	0.5	0.0039	>0.5	0.5	0.5	0.0078	>0.5	>0.5	>0.5	>0.5	>0.5	>0.5	>0.5	>0.5

## Data Availability

The data presented in this study are available from the corresponding author upon request.

## Supplementary Materials

The following supporting information can be downloaded at: https://www.mdpi.com/article/10.3390/ijms26093930/s1.

## List of Abbreviations and Symbols

AgNPs	Silver nanoparticles
IL	Interleukin
MRS	Methicillin-resistant Staphylococcus
WHO	World Health Organization
SCN	Coagulase-negative Staphylococcus
TLR	Toll-like receptor
Antibiotics	
AK	Amikacin
AMC	Amoxicillin
AMP	Ampicillin
ATM	Aztreonam
C	Chloramphenicol
CAZ	Ceftazidime
CIP	Ciprofloxacin
CT	Colistin
CXM	Cefuroxime
DA	Clindamycin
E	Erythromycin
FEP	Cefepime
FOX	Cefoxitin
GN	Gentamicin
IMP	Imipenem
LEV	Levofloxacin
LZD	Linezolid
MEM	Meropenem
NET	Netilmicin
P	Penicillin
SXT	Trimethoprim sulfamethoxazole
TEC	Teicoplanin
TIM	Ticarcillin-clavulanate
TOB	Tobramycin
