# Supplementary material for: Silver Nanoparticles Functionalized with Polymeric Substances to Reduce the Growth of Planktonic and Biofilm Opportunistic Pathogens
Source: Int J Mol Sci. 2025 Apr 22;26(9):3930. doi: 10.3390/ijms26093930 (PMC12071338; doi:10.3390/ijms26093930)
Supplement: Supplementary file 1 [file ijms-26-03930-s001.zip › ijms-3570729-supplementary.pdf]

Table S1: Antibiotic resistance profiles of clinical bacterial isolates obtained from Romanian hospitals. The isolates include Escherichia coli, Klebsiella pneumoniae, Pseudomonas aeruginosa, and Staphylococcus spp., all recovered from difficult-to-treat infections such as urinary tract infections, respiratory samples, and skin lesions. Antibiotic susceptibility was assessed using standard disc diffusion or MIC methods, and resistance patterns were interpreted according to CASFM/EUCAST guidelines.

**no**	**bacterial strains**	**source of sample**	**IMI**	**MEM**	**FOX**	**CAZ**	**ATM**	**AMC**	**CRO**	**PRL**	**F**	**AK**	**CN**	**TOB**	**CIP**	**NOR**	**LEV**	**MXF**	**SXT**	**W**	**TGC**	**TZP**	**ERT**	AML	TIC			
1	*Escherichia coli*	urine	S	S	S	I	R	I	R	R	S	S	S	S	R	R	R	R	R	R	S	S	S	S	R			
10	*Escherichia coli*	urine	S	S	S	S	S	R	S	R	S	S	R	R	R	R	R	R	S	S	S	S	S	S	R			
11	*Escherichia coli*	urine	S	S	S	S	S	I	S	S	S	S	S	S	S	S	S	S	S	S	S	S	S	S	S			
13	*Escherichia coli*	urine	S	S	S	S	S	R	S	R	S	S	S	S	S	S	S	S	R	R	S	S	S	S	R			
14	*Escherichia coli*	urine	S	S	S	S	I	R	R	R	S	R	S	R	S	S	S	S	R	R	S	S	S	R	R			
16	*Escherichia coli*	urine	S	S	S	S	S	I	S	R	S	S	S	S	S	S	S	S	R	R	S	S	S	R	R			
17	*Escherichia coli*	urine	S	S	R	S	S	R	S	S	S	S	S	S	R	R	R	R	S	S	S	S	S	R	S			
19	*Escherichia coli*	urine	S	S	S	S	S	R	S	R	S	S	S	S	S	S	S	S	R	R	S	S	S	R	R			
20	*Escherichia coli*	urine	S	S	S	S	S	I	I	S	S	S	S	S	S	S	S	S	R	R	S	S	S	S	S			
21	*Escherichia coli*	urine	S	S	R	S	R	R	R	R	S	S	S	R	R	R	R	R	R	R	S	S	S	R	R			
27	*Escherichia coli*	urine	S	S	S	S	S	I	S	R	S	S	S	S	S	S	S	S	R	R	S	S	S	R	R			
3	*Klebsiella pneumoniae*	urine	S	S	S	S	S	I	S	S	S	S	S	S	S	S	S	S	S	S	S	S	S	R	R			

8	*Klebsiella pneumoniae*	urine	S	S	S	S	S	I	S	R	S	S	S	S	I	R	S	R	S	R	S	S	S	R	R			
24	*Klebsiella pneumoniae*	urine	S	S	R	R	R	R	R	R	S	S	R	R	R	R	R	R	R	R	S	R	R	R	R			
160	*Klebsiella pneumoniae*	urine	S	S	S	S	S	R	S	R	R	S	S	R	R	R	R	R	R	R	S	R	R	R	R			
168	*Klebsiella pneumoniae*	urine	R	R	R	R	R	R	R	R	S	R	R	R	R	R	R	R	R	R	S	R	R	R	R			
172	*Klebsiella pneumoniae*	urine	S	S	R	S	S	R	S	S	R	S	S	S	R	R	S	S	S	S	S	S	S	R	R			
180	*Klebsiella pneumoniae*	urine	R	R	R	R	R	R	R	R	R	S	R	S	R	R	R	R	S	S	S	R	R	R	R			
289	*Klebsiella pneumoniae*	urine	S	S	S	S	S	I	S	S	S	S	S	S	S	S	S	S	S	S	S	S	S	R	R			
296	*Klebsiella pneumoniae*	urine	S	S	S	S	S	S	S	R	S	S	S	S	R	R	R	R	S	S	S	S	S	R	R			
302	*Klebsiella pneumoniae*	urine	S	S	S	S	S	I	S	R	S	S	S	S	S	S	S	S	R	R	S	S	S	R	R			
305	*Klebsiella pneumoniae*	urine	R	R	R	R	S	R	R	R	R	R	R	R	R	R	R	R	R	R	S	R	R	R	R			
			**CAZ**	**FEP**	**ATM**	**TIC**	**LEV**	**CIP**	**AK**	**TOB**	**PRL**	**TZP**																
12	*Pseudomonas aeruginosa*	urine	I	I	I	I	I	I	S	S	S	S																
15	*Pseudomonas aeruginosa*	urine	I	I	I	I	I	I	S	S	S	S																
16	*Pseudomonas aeruginosa*	urine	I	I	I	I	I	I	S	S	S	S																
18	*Pseudomonas aeruginosa*	urine	I	R	I	I	I	I	S	S	S	S																
22	*Pseudomonas aeruginosa*	urine	I	I	I	I	I	I	S	S	S	S																

29	*Pseudomonas aeruginosa*	urine	R	R	I	R	I	I	S	S	S	S																
31	*Pseudomonas aeruginosa*	urine	I	I	I	I	I	I	S	S	S	S																
34	*Pseudomonas aeruginosa*	urine	I	I	I	R	R	R	S	S	R	R																
35	*Pseudomonas aeruginosa*	urine	I	I	I	I	I	I	S	S	S	S																
37	*Pseudomonas aeruginosa*	urine	I	I	I	I	I	I	S	S	S	S																
39	*Pseudomonas aeruginosa*	urine	I	I	I	I	I	I	S	S	S	S																
40	*Pseudomonas aeruginosa*	urine	I	I	I	I	I	I	S	S	R	S																
41	*Pseudomonas aeruginosa*	urine	R	I	I	I	I	I	S	S	S	S																
42	*Pseudomonas aeruginosa*	urine	R	I	I	I	I	I	S	S	S	S																
43	*Pseudomonas aeruginosa*	urine	I	R	I	I	I	I	S	S	S	S																
44	*Pseudomonas aeruginosa*	urine	I	I	I	I	I	I	S	S	S	S																
45	*Pseudomonas aeruginosa*	urine	I	I	I	I	I	I	S	S	S	S																
46	*Pseudomonas aeruginosa*	urine	R	I	I	I	I	I	S	S	S	S																
47	*Pseudomonas aeruginosa*	urine	R	I	I	I	I	I	S	S	S	S																
48	*Pseudomonas aeruginosa*	urine	R	I	I	I	I	I	S	S	S	S																
49	*Pseudomonas aeruginosa*	urine	I	R	I	I	I	I	S	S	R	S																

52	*Pseudomonas aeruginosa*	urine	I	I	I	I	I	I	S	S	S	S																
			**TOB**	**SXT**	**FD**	**MUP**	**NOR**	**NET**	**K**	**CN**	**TGC**	**RD**	**W**	**FOT**	**LZD**	**MH**	**QD**	**FOX**	**P**	**DA**	**E**	**C**	**TE**	**F**	**CIP**	**LEV**	**MXF**	**OFX**
9	*Staphylococcus aureus*	urine	S	S	S	S	S	S	S	S	S	S	S	S	S	S	S	S	R	R	R	S	S	S				
13	*Staphylococcus aureus*	pulmonary sputum from cystic fibrosis	S	S	S	S	S	S	R	S	S	S	S	S	S	S	S	S	R	R	R	S	R	S				
14	*Staphylococcus aureus*	nasal exudate	S	S	S	S	S	S	R	S	S	S	S	S	S	S	S	R	R	S	R	S	R	S				
25	*Staphylococcus aureus*	acnee	S	S	S	S	S	S	S	S	S	S	S	S	S	S	S	S	S	S	S	S	R	S				
27	*Staphylococcus aureus*	nasal exudate	S	S	R	S	S	S	R	S	S	S	S	S	S	S	S	R	R	S	S	S	R	S				
42	*Staphylococcus aureus*	purulent secretion	S	S	S	I	S	S	S	S	S	S	S	S	S	R	S	S	R	S	I	S	R	S				
47	*Staphylococcus aureus*	pustules scalp	S	S	S	S	S	S	S	S	S	S	S	S	S	S	S	S	R	S	S	S	S	S				
51	*Staphylococcus aureus*	nasal exudate	S	R	S	S	R	S	S	S	S	S	R	S	R	S	S	S	R	S	R	S	R	S	R	R	R	R
59	*Staphylococcus aureus*	purulent secretion	S	I	S	S	S	S	S	S	S	S	S	S	S	S	S	S	S	S	S	S	S	S				
63	*Staphylococcus aureus*	purulent secretion	S	S	S	S	S	S	S	S	S	S	S	S	S	R	S	S	S	S	S	S	R	S				
65	*Staphylococcus aureus*	osteomyelitis	S	S	S	S	S	S	R	S	S	S	S	S	S	S	S	R	R	S	R	S	S	S				
66	*Staphylococcus aureus*	acnee	S	S	S	S	S	S	R	S	S	S	S	S	S	S	S	S	R	I	R	S	R	S				
91	*Staphylococcus epidermidis*	acne	S	S	S	S	S	S	S	S	S	S	S	S	S	S	S	S	R	S	R	S	S	S				

107	*Staphylococcus epidermidis*	hidrosadenitis	S	S	S	S	S	S	S	S	S	S	S	S	S	S	S	S	S	S	S	S	S	S				
109	*Staphylococcus epidermidis*	acne	S	S	S	S	S	S	S	S	S	S	S	S	S	S	S	S	R	S	S	S	S	S				
177	*Staphylococcus epidermidis*	acne	R	S	S	S	S	S	R	R	S	S	R	S	S	S	S	S	R	R	R	S	S	S				
182	*Staphylococcus epidermidis*	acne	S	S	S	S	S	S	S	S	S	S	S	S	S	S	S	S	S	R	R	S	S	S				
186	*Staphylococcus epidermidis*	acne	S	S	S	S	S	S	S	S	S	S	S	S	S	S	S	S	R	R	R	S	S	S				
206	*Staphylococcus lugdunensis*	acne	S	S	S	S	S	S	S	S	S	S	I	S	S	S	S	S	R	S	S	S	S	S				
209	*Staphylococcus lugdunensis*	purulent secretion	S	S	S	S	S	S	S	S	S	S	I	S	S	S	S	S	S	S	S	S	S	S				
216	*Staphylococcus lugdunensis*	purulent secretion	S	S	S	S	S	S	S	S	S	S	I	S	S	S	S	S	R	R	R	S	R	S				
223	*Staphylococcus lugdunensis*	acne	S	S	S	I	S	S	S	S	S	S	S	R	S	S	S	S	R	S	S	S	S	S				
224	*Staphylococcus lugdunensis*	purulent secretion	S	S	S	S	S	S	R	S	S	S	R	S	S	S	S	S	R	S	S	S	R	S				
226	*Staphylococcus lugdunensis*	acne	S	S	S	S	S	S	S	S	S	S	R	S	S	S	S	S	R	S	S	S	S	S				
